# New Solid Phase Synthesis of Distamycin Analogues 

**DOI:** 10.3390/molecules16043066

**Published:** 2011-04-11

**Authors:** Drozdowska Danuta

**Affiliations:** Department of Organic Chemistry, Medical University, Mickiewicza 2A Str., 15-222 Białystok, Poland; Email: danuta.drozdowska@umwb.edu.pl; Tel.: +48 85 7485684; Fax: +48 85 7485416

**Keywords:** combinatorial chemistry, solid phase synthesis, distamycin analogues

## Abstract

A novel and straightforward solid phase synthesis of distamycin analogues containing benzene units has been developed.

## 1. Introduction

**Figure 1:**
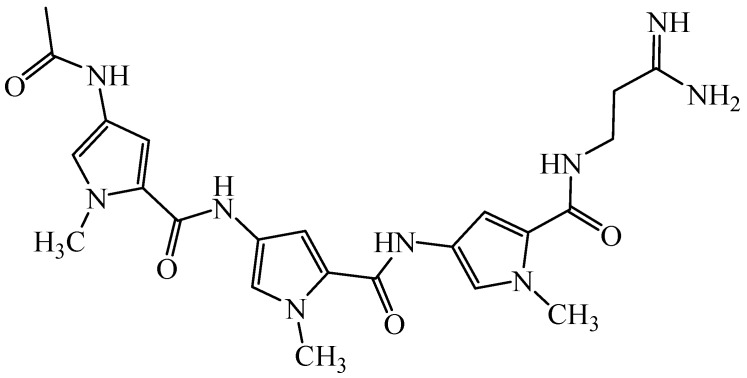
Structure of distamycin A.

Distamycin A (Figure 1) is natural antibiotic with anticancer activity. This compound is one of the most extensively studied members of class of molecules that can bind reversibly in the minor groove of duplex DNA, by hydrogen bonds, van der Waals contacts and electrostatic interactions, with high specificity for regions containing A-T base pairs [1].The molecule of distamycin is oligopeptide constructed with 4-amino-1-methylpyrrole acid moieties and strong basic side chains, possessing isohelical shape to minor groove DNA [2,3].

This antibiotic is too toxic to find application in cancer therapy [4], but the manner in which distamycin binds to DNA has inspired the search for compounds with similar mode of action; some of them are being tested in clinical trials [5]. The group of synthetic oligopeptides, designed based on the pattern of described antibiotic and selective to specific base pairs of DNA, obtained the name “lexitropsins”[4]. Distamycins and other lexitropsins display both antiviral and antibiotic activity. Some of them show interesting antiprotozoal activity related to the ability to reversibly bind to the minor groove of DNA with a high selectivity for TA-rich sequences. For these reasons a lot of various distamycin analogues and conjugates have been synthesized, also with application of combinatorial chemistry methods. 

Baird and Dervan described solid phase synthesis of polyamides containing imidazole and pyrrole amino acids using *tert*-butyloxycarbonyl-protection startegy [6]. Boger and co-workers used above strategy to design a library of 2,640 compounds inspired by the structure of distamycin A. They applied solution-phase synthesis and acid/base liquid – liquid extraction techniques to isolate the compounds [7]. Brucoli and others employed SynPhase Lanterns to prepare a library of 72 novel distamycin analogues, where one of pyrrole rings was replaced by biaryl motifs [8]. Baraldi’s group synthesized and evaluated series α-methylene-γ-butyrolactone-lexitropsin hybrids [9], while Bhattacharya and Thomas reported the first example of cholesterol-conjugated distamycin analogues, which retain their strong binding capacity to double-stranded (ds)-DNA [10]. Fluorescent distamycins were also synthesized, which enable to study of the kinetics of polyamide-DNA interactions and monitoring of their cellular distribution [11]. Such molecules report the physical details of DNA binding sites, e.g. polyamide part of the distamycin-porphyrin conjugates was found to bind to the minor groove of DNA with preference for A-T rich sequences, while the fluorescent porphyrin fragment exhibited intercalation and the non-specific electrostatic interaction with the phosphate groups of DNA [12]. 

Synthesis and evaluation of distamycin analogues without the leading amide unit at the N-terminus demonstrated that a hydrogen bond donors or acceptor atom *per se* at the N-terminus is not essential for their DNA binding, however a minimum of three pyrrole carboxamide units is necessary for the onset of DNA binding [13,14]. Interestingly, intense induced circular dichroism (ICD) spectra were obtained not only with AT-rich DNA tracts, but also with poly d(GC), even in such salt concentrations of the solution, at which poly d(GC) is likely to exist in the Z conformation [15,16]. These results confirmed the involvement of polyamide-based minor groove binders in gene regulation processes and showed that thus subtle modifications in the ligand molecular structure can have dramatic effect on their DNA binding properties. 

For improved DNA recognition Valík and other synthesized ‘head-to-head’ oligo-N-methylpyrrole peptide dimers linked by a methanol[1,5]diazocin scaffold. DNA binding study in racemic, as well as chiral fashion, showed that novel dimers prefer AT sequences, but show higher affinity to poly(dC-dG)_2_ than distamycin. Moreover, the directionality can be controlled by the stereochemistry of used linker [17]. Ghosh and co-authors designed photoisomerizable azobenzene-distamycin conjugates in which two distamycin units were linked via electron-rich alkoxy or electron withdrawing carboxamide moieties with the azobenzene core. Duplex DNA binding abilities of these conjugates were found to directly depend upon the nature and length of the spacer, the location of protonatable residue, and the isomeric state of the conjugate [18]. 

Distamycin-like minor groove binders reach the cell nucleus with difficulty due to their poor membrane permeability. Synthetic tripyrrole-octa-arginine conjugates investigated by Vázquez and others are capable to rapid localization in the cell nuclei with low nanomolecular affinity, targeting specific DNA sites that contain AT-rich tracts. Moreover there were observed significant synergistic transport effects between the minor-groove binding tripyrrole unit and the octa-arginine peptide, which cooperate in localizing the hybrid molecules in the cell nuclei [19]. In the field of drug delivery using distamycin derivatives, selected sequences of oligodeoxyribonucleotides (ODNs) were conjugated efficiently with distamycin-based peptides containing reactive cysteine and oxyamine functionalities at the C-terminus. The method described is time-saving and it allows investigating of DNA based diagnostic properties of antigens or therapeutic ODNs by enhancing its target-binding properties, cellular uptake, or exonuclease stability or can be used as an in situ hybridization probe [20]. 

Since small molecules that target G-quadruplexes have been found to be effective telomerase inhibitors, the identification of new specific ligands for G-quadruplexes is emerging as a promising approach to develop new anticancer drugs. Distamycin A is known to bind to AT-rich sequences of duplex DNA, but it has recently been shown to interact also with four-stranded parallel DNA quadruplexes [21,22]. This finding has stimulated syntheses of distamycin polyamides targeting G-quadruplex DNA. For example, Moore and co-workers have synthesized a number of oligopyrrole by solid-state methods and investigated their interactions with a human intramolecular G-quadruplex [23]. As it can be seen, there are a lot of different applications for distamycin and its analogues, therefore new synthesis methods are still in demand.

Most of described approaches to solid phase synthesis of distamycin analogues are based on traditional peptide strategy. Here, a different way of solid phase synthesis, inspired by earlier investigation in the field of carbocyclic lexitropsin synthesis is presented. Described in this paper chemistry is based on the procedure adopted previously to syntheses of carbocyclic minor groove binders [24]. This strategy uses aromatic nitro compounds – amines and acyl chlorides – to build carbocyclic oligoamides. The precursor nitro group of first substrate is reduced, meanwhile obtained amine, in the next step of the process, undergoes acylation with acyl chloride containing the next nitro group in the presence of DMAP at room temperature. 

Over the last few years the synthesis and biological evaluation of derivatives containing benzene in place of N-methylpyrrole rings have been reported by me and others. These carbocyclic compounds bind to AT sequences less strongly than distamycin, however these compounds show sequence selectivity [25]. Carbocyclic lexitropsins are readily available, can be modified easily, and are stable under most experimental conditions. All of the tested carbocyclic potential minor groove binders showed the antiproliferative and cytotoxic effects against MCF-7 and/or MBA-MB-231 breast cancer cell lines [26,27]. The carbocyclic analogues of distamycin with unsubstituted N-terminal group NH_2 _inhibited *in vitro* activity of topoisomerase I and II [28] and amidolytic activity of proteolytic enzymes, such as plasmin or urokinase [29]. The carbocyclic lexitropsins investigated so far were not sufficiently active to be used as agent in breast cancer therapy but the application of them as potential carriers of strong acting elements was also examined. For example, derivatives with N-terminal chlorambucyl group also exhibited activity in cultured breast cancer MCF -7 [30].

Solid phase synthesis methodology is able to increases both the number and complexity of new compound, which can be synthesized. Here we present a new method of solid-phase synthesis of distamycin derivatives, in continuation of searching more selective DNA-binding molecules, based on distamycin as a lead molecule, 

## 2. Results and Discussion

**Scheme 1:**
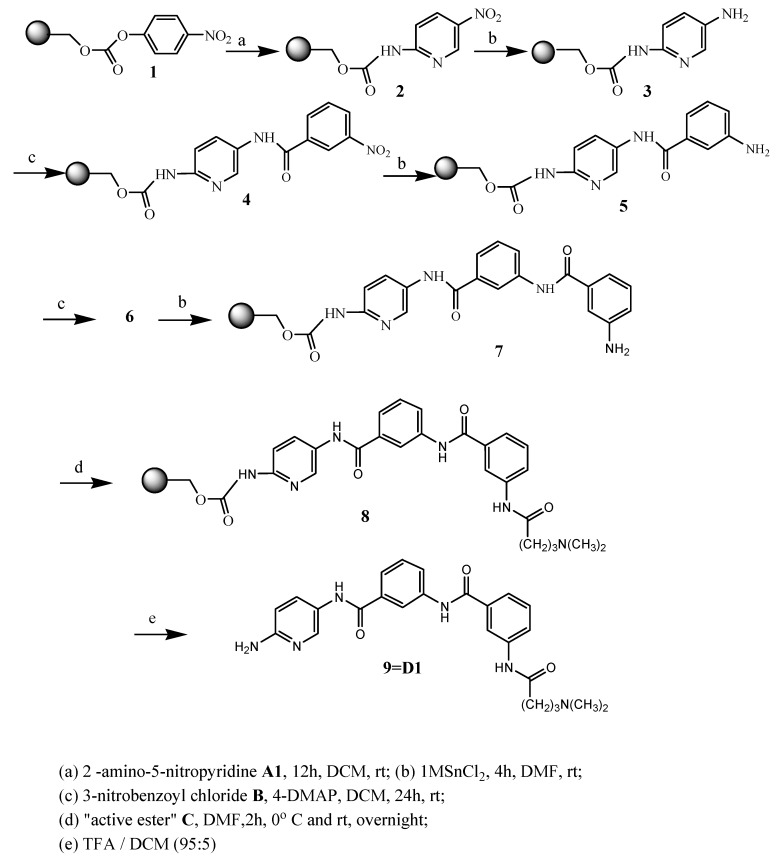
Solid phase synthesis of distamycin analogues.

The preparation of distamycin derivatives was performed with *p*-nitrophenyl carbonate Wang resin **1**, in the same way as is shown for compound **D1** in Scheme 1. Resin-bound *p*-nitrophenyl carbonate ester reacts readily with amines to provide the corresponding resin-bound carbamates. Six commercially available aromatic amino-nitro compounds **A1-A6** (see Table 1) were used in the first step of the described synthesis. After grafting of nitroamines to resin, reduction of the nitro group of **2** was carried out by using the dihydrate of tin (II) chloride in DMF.

To confirm that aromatic amine groups were obtained, the chloranil test has been used [31]. Acylation of amine **3 **by use of 3-nitrobenzoyl chloride **B** in the presence of DMAP in methylene chloride at room temperature produced the resin-bound nitro compound **4**. Yet another reaction of reduction led to obtainment the compound **5**, which after next acylation with chloride **B** resulted with obtainment of nitro compound **6**. Repeated reduction of nitro group gave amino compound **7**. 

**Scheme 2:**
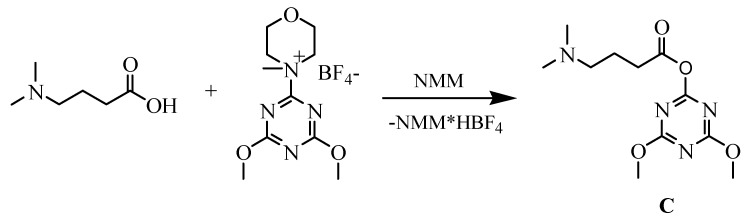
Synthesis of triazine active ester **C** from 4-dimethylaminobutyric acid.

The advantage of exchange of the amidinium moiety (normally presented in distamycin) by the dimethylamino group was described earlier [32]. The compounds containing a modified terminus are chemically stable, not hygroscopic and are easy to handle. To introduce similar fragment in new distamycin analogues, distinctive only in amide bond direction, 4-dimethylaminobutyric acid was used. Activation of this acid for the formation of amide bond was a key step in our synthesis, so we used the new generation of triazine based coupling reagent (4-(4,6-dimethoxy-1,3,5-triazin-2-yl)-4-methylmorpholinium tetrafluoroborate) [33] and prepered triazine “superacrive ester” **C**, as shown in Scheme 2.

The activated acid **C** was added to intermediate product **7** and final resin-bond analogue of distamycin **8** was obtained. Cleavage by 95% trifluoroacetic acid in dichloromethane gave the desired compound in satisfactory yield. The structure, analytical and spectrometric data are presented in Table 1.

**Table 1:** Analytical and spectral data of the synthesized compounds **D1**-**D6**.

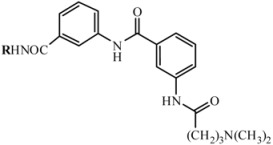						
No.	Substrate A	Fragment R	Yield [%]	R_t_	Exact MassFormula	[M+H]^+^
**D1**	**A_1_**	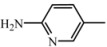	66	18.23	460.5	459.5
	2-amino-5-nitropyridine				C_25_H_28_N_6_O_3_	
**D2**	**A_2_**	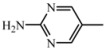	55	18.29	461.5	460.4
	2-amino-5-nitropyrimidine				C_24_H_27_N_7_O_3_	
**D3**	**A_3_**	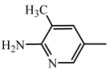	47	18.27	474.6	473.5
	2-amino-3-methyl-5-nitropyridine				C_26_H_30_N_6_O_3_	
**D4**	**A_4_**	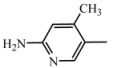	49	18.24	474.6	473.5
	2-amino-4-methyl-5-nitropyridine					
					C_26_H_30_N_6_O_3_	
**D5**	**A_5_**	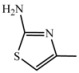	58	18.33	466.5	465.4
	2-amino-5-nitrotiazole				C_23_H_26_N_6_O_3_S	
**D6**	**A_6_**	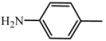	73	18.18	459.5	458.4
	3-nitroaniline				C_26_H_29_N_5_O_3_	

## 3. Experimental

All reagents were purchased from Lancaster, Fluka, Merck, Alfa Aesar or Iris Biotech GmBH and used without further purification. 4-(4,6-dimethoxy-1,3,5-triazin-2-yl)-4-methylmorpholinium tetra-fluoroborate was prepared in Institute of Organic Chemistry Technical University in Łódź. Dichloromethane (DCM) and dimethylformamide (DMF) were stored under 4Å molecular sieves. ^1^H-NMR and ^13^C-NMR spectra were recorded on a Bruker AM 200 spectrometer using TMS as internal standard; chemical shifts δ are reported in ppm. The spectra were recorded at room temperature. LC-MS spectra were recorded on Bruker Daltonics Esquire 6000 instrument with electrospray ionization (ESI). A Shimadzu LC-10A system was used for analytical HPLC (Phenomenex C18, Jupiter 90A, 4 micron, 250 x 10 mm; Phenomenex C18, Jupiter 300A, 5 micron, 250 x 4 mm; solvents: A, 0.1% aqueous TFA; B, 0.1% TFA in acetonitrile, gradient 0% B to 60% B in A in 30 min, flow rate 1 ml/min, monitored at 220 nm). 

### General Procedure

The solid-phase syntheses of the new compounds are shown in Scheme 1. 4-Nitrophenyl Wang resin **1** (0.5 g; 0.41mmol; 0.81 mmol/g) was suspended and swelled for 10 min in dry DCM (10 mL), then the resin was treated with nitroamines (**A1-A6**; 4 mmol) dissolved in DCM (20 mL) and pyridine (177.22 μl; 2.2 mmol). Then the mixture was stirred for 12 h. The resin **2** was washed five times with DCM (20 mL) and three times with DMF (20 mL). Nitro group reduction of **2** was then carried out with the dihydrate of tin (II) chloride in DMF (1M; 10 mL) during 4 h at room temperature. The resin **3** was filtered off and then washed five times with DMF (20 mL) and three times with DCM (20 mL). Then 3-nitrobenzoyl chloride **B **(0.304 g; 1.64 mmol; 4 eq) with DMAP (0.0025 g; 0.0205 mmol) were dissolved in DCM (20 mL) and added to resin **3**. The stirring was continued at room temperature overnight, which gives the resin-bound nitro compound **4**. 

The next washing and synthetic procedures were the same as for compounds **2** and **3**. The treatment with 1M SnCl_2_ was repeated once again to obtain the compound **5**, which after acylation with chloride **B** afforded next nitro compound **6**. Repeated reduction of nitro group gave amino compound **7**. 

To prepare “superactive ester” **C** with 4-dimethylaminobutyric acid, this amino acid (0.137 g; 0.82 mmol) and NMM (902 μL; 0.82 mmol) were added to a vigorously stirred solution of 4-(4,6-dimethoxy-1,3,5-triazin-2-yl)-4-methylmorpholinium tetrafluoroborate (0.269 g; 0.82 mmol) in DMF (20 mL), cooled to 0 ^o^C. The stirring was continued for an additional 2 h in 0 ^o^C, after which time the resin **7** was added ,and the mixture stirred for an additional 2h at 0 ^o^C and overnight at room temperature. The resin **8** was washed with DCM (5 x 10 mL), dried and then treated with TFA/DCM (95:5). Evaporation of the solvents and yielded the products as glaze solid.

*N-(6-aminopyridin-3-yl)-3-[3-(4-dimethylaminobutyrylamino)benzamido]benzamide* (**D1**). Starting material **A1** (0.226 g). Product **D1** (0.125 g, 66%). ^1^H-NMR (DMSO-D_6_) δ = 10.36 (s,1H, NH), 10.04 (s,1H, NH), 8.27 (t,1H, NH), 8.13-6.74 (m, 10H), 4.72-4.26 (bs, 2H, NH_2_), 2.91 (d, 2H, CH_2_), 2.72 (d, 2H, CH_2_), 2.57 (s, 6H, CH_3_), 2.50 (m, 2H, CH_2_). ^13^C-NMR (DMSO-D_6_) δ = 165.76 (CONH), 164.98 (CONH), 162.30 (CONH), 153.70 (C), 139.29 (C), 136.50 (C), 135.83 (C), 130.69 (C), 129.17 (CH), 128.51 (CH), 122.99 (CH), 122.36 (CH), 122.20 (CH), 120.72 (CH), 120.73 (C), 119.85 (CH), 119.68 (CH), 118.75 (CH), 116.23 (CH), 114.97 (CH), 41.50 (CH_2_), 39.52 (CH_3_), 35.76 (CH_2_), 30.76 (CH_2_).

*N-(2-aminopyrimidin-5-yl)-3-[3-(4-dimethylaminobutyrylamino)benzamido]benzamide* (**D2**). Starting material **A2** (0.225 g). Product **D2** (0.104 g, 55%). ^1^H-NMR (DMSO-D_6_) δ = 10.37 (s,1H, NH), 10.04 (s,1H, NH), 8.27 (t,1H, NH), 8.13-6.72 (m, 11H), 5.32-4.50 (bs, 2H, NH_2_), 2.88 (d, 2H, CH_2_), 2.72 (d, 2H, CH_2_), 2.57 (s, 6H, CH_3_), 2.50 (m, 2H, CH_2_). ^13^C-NMR (DMSO-D_6_) δ = 165.84 (CONH), 164.96 (CONH), 162.30 (CONH), 153.70 (C), 139.20 (C), 136.50 (C), 135.83 (C), 130.69 (C), 129.15 (CH), 128.51 (CH), 123.06 (CH), 122.99 (CH), 122.20 (CH), 120.71 (CH), 119.85 (CH), 119.68 (CH), 118.65 (C), 115.88 (CH), 114.98 (CH), 41.50 (CH_2_), 39.52 (CH_3_), 35.76 (CH_2_), 30.76 (CH_2_).

*N-(6-amino-5-methylpyridin-3-yl)-3-[3-(4-dimethylaminobutyrylamino)benzamido]benzamide* (**D3**). Starting material **A3** (0.251 g). Product **D3** (0.91 g, 47%). ^1^H-NMR (DMSO-D_6_) δ = 10.50 (s,1H, NH), 10.03 (s,1H, NH), 8.27 (t,1H, NH), 7.98-6.71 (m, 10H), 5.45-4.58 (bs, 2H, NH_2_), 2.88 (d, 2H, CH_2_), 2.78 (d, 2H, CH_2_), 2.60(s, 3H, CH_3_), 2.58 (s, 6H, CH_3_), 2.55 (m, 2H, CH_2_). ^13^C-NMR (DMSO-D_6_) δ = 165.64 (CONH), 164.97 (CONH), 162.30 (CONH), 153.70 (C), 142.46 (C), 139.26 (C), 135.86 (C), 135.60 (C), 130.69 (C), 129.25 (CH), 128.52 (CH), 123.08 (CH), 123.01 (C), 122.41 (CH), 122.20 (CH), 120.58 (CH), 119.87 (CH), 119.47 (CH), 116.80 (CH), 114.97 (CH), 41.50 (CH_2_), 39.52 (CH_3_), 39.04 (CH_3_), 35.76 (CH_2_), 30.76 (CH_2_).

*N-(6-amino-4-methylpyridin-3-yl)-3-[3-(4-dimethylaminobutyrylamino)benzamido]benzamide* (**D4**). Starting material **A4** (0.251 g). Product **D4** (0.095 g, 49%). ^1^H-NMR (DMSO-D_6_) δ = 10.36 (s,1H, NH), 10.03 (s,1H, NH), 8.27 (t,1H, NH), 7.97-6.71 (m, 10H), 4.92-4.18 (bs, 2H, NH_2_), 2.89 (d, 2H, CH_2_), 2.78 (d, 2H, CH_2_), 2.62(s, 3H, CH_3_), 2.58 (s, 6H, CH_3_), 2.55 (m, 2H, CH_2_). ^13^C-NMR (DMSO-D_6_) δ = 165.79 (CONH), 164.95 (CONH), 162.29 (CONH), 153.69 (C), 139.20 (C), 135.86 (C), 135.79 (C), 130.69 (C), 129.14 (CH), 128.49 (CH), 122.99 (CH), 122.35 (CH), 122.19 (CH), 122.09 (C) 120.72 (CH), 119.84 (CH), 118.52 (CH), 116.03 (CH), 114.96 (CH), 41.50 (CH_2_), 39.52 (CH_3_), 39.04 (CH_3_), 35.76 (CH2), 30.76 (CH_2_).

*N-(2-aminothiazol-4-yl)-3-[3-(4-dimethylaminobutyrylamino)benzamido]benzamide* (**D5**). Starting material **A5** (0.235 g). Product **D5** (0.111 g, 58%). ^1^H-NMR (DMSO-D_6_) δ = 10.35 (s,1H, NH), 10.04 (s,1H, NH), 8.27 (t,1H, NH), 7.98-6.72 (m, 9H), 4.57-3.92 (bs, 2H, NH_2_), 2.81 (d, 2H, CH_2_), 2.78 (d, 2H, CH_2_), 2.57 (s, 6H, CH_3_), 2.50 (m, 2H, CH_2_). ^13^C-NMR (DMSO-D_6_) δ = 165.83 (C), 165.09 (CONH), 164.95 (CONH), 162.29 (CONH), 153.69 (C), 139.20 (C), 136.50 (C), 135.82 (C), 130.69 (C), 129.13 (CH), 128.53 (CH), 123.09 (CH), 122.98 (CH), 122.20 (CH), 120.72 (CH), 119.92 (CH), 119.65 (CH), 93.35 (CH), 41.50 (CH_2_), 39.52 (CH_3_), 35.76 (CH2), 30.76 (CH_2_).

*N-(4-aminophenyl)-3-[3-(4-dimethylaminobutyrylamino)benzamido]benzamide* (**D6**). Starting material **A6** (0.226 g). Product **D6** (0.137 g, 73%). ^1^H-NMR (DMSO-D_6_) δ = 10.36 (s, 1H, NH), 10.04 (s, 1H, NH), 8.26 (t, 1H, NH), 7.96-6.73 (m, 12H), 4.57-4.12 (bs, 2H, NH_2_), 2.87 (d, 2H, CH_2_), 2.78 (d, 2H, CH_2_), 2.52 (s, 6H, CH_3_), 2.40 (m, 2H, CH_2_). ^13^C-NMR (DMSO-D_6_) δ = 165.78 (CONH), 164.98 (CONH), 162.31 (CONH), 153.70 (C), 139.29 (C), 135.86 (C), 135.81 (C), 130.70 (C), 129.19 (CH), 128.52 (CH), 123.00 (C), 122.38 (CH), 122.06 (CH),120.03 (CH), 119.96 (CH), 119.86 (CH), 118.78 (CH), 118.72 (CH), 116.19 (CH), 114.98 (CH).

## 4. Conclusions

The approach presented here is efficient and practical. A simple and general procedure for solid phase synthesis of distamycin analogues, using the different strategy than most of described approaches based on traditional synthesis of peptides, has been developed. The elaborated method of solid phase synthesis of amide bonds, with the use of nitro group, allows building carbocyclic oligoamides both with any desired number of benzene rings and the different heterocyclic rings. Our method makes it possible to parallel syntheses of many new compounds, permitting automation of the process. The presented method of distamycin analogues preparation can use several different aromatic nitro amines, as well as acyl chlorides with nitro group, so it allows obtainment of a lot of new compounds based on distamycin A as the lead molecule. The effective introduction of 4-dimethylaminobutyric acid fragment, in the last step of synthesis, permit the synthetic procedure to obtain enough flexibility such that it would accommodate introduction of other fragments, for further modulation of the DNA binding properties. Such work, toward the synthesis of novel minor groove binders with different building units, now is carried out in our laboratory.

## Supplementary Materials

Supplementary File 1
