# Drozdowska D., New Solid Phase Synthesis of Distamycin Analogues. *Molecules*, 2011, *16*, 3066-3076

**DOI:** 10.3390/molecules16075753

**Published:** 2011-07-06

**Authors:** Danuta Drozdowska

**Affiliations:** Department of Organic Chemistry, Medical University, Mickiewicza 2A Str., 15-222 Białystok, Poland; E-Mail: danuta.drozdowska@umwb.edu.pl; Tel.: +48 85 7485684; Fax: +48 85 7485416

The author wishes to make the following correction to this paper [1]:

The correct author’s name is: Danuta Drozdowska.
