# Supplementary material for: New Solid Phase Synthesis of Distamycin Analogues
Source: Molecules. 2011 Apr 11;16(4):3066–76. doi: 10.3390/molecules16043066 (PMC6260608; doi:10.3390/molecules16043066)
Supplement: Supplementary File 1 [file molecules-16-03066-s001.pdf]

Molecules **2011**, *16*, 5753; doi:10.3390/molecules16075753

OPEN ACCESS

molecules

ISSN 1420-3049

www.mdpi.com/journal/molecules

Correction

Drozdowska D., New Solid Phase Synthesis of Distamycin Analogues. *Molecules*, 2011, *16*, 3066-3076

Danuta Drozdowska

Department of Organic Chemistry, Medical University, Mickiewicza 2A Str., 15-222 Białystok, Poland; E-Mail: danuta.drozdowska@umwb.edu.pl; Tel.: +48 85 7485684; Fax: +48 85 7485416

Received: 20 June 2011 / Published: 6 July 2011

The author wishes to make the following correction to this paper [1]:

The correct author's name is: Danuta Drozdowska.

Reference

1. Drozdowska, D. New Solid Phase Synthesis of Distamycin Analogues. *Molecules* **2011**, *16*, 3066-3076.

© 2011 by the authors; licensee MDPI, Basel, Switzerland. This article is an open access article distributed under the terms and conditions of the Creative Commons Attribution license (http://creativecommons.org/licenses/by/3.0/).
